# Hospital Problems in India

**Published:** 1920-10-30

**Authors:** 


### HOSPITAL PROBLEMS IN INDIA.

### Village Sanitation.

A conference of the headmen of villages was held at the Government farm near Bacca. This conference has met annually since 1917, and the value of the work done by it is proved by the fact that out of thirty resolutions passed by the members at the 1917 and 1918 sessions no less than twelve were embodied in the Village Self-Government Act of 1919, such as the desirability of providing vaccination free to every resident in each group or union of villages. Some of the unions have tried this with great success. Another was the possibility of two or more unions co-operating to subsidise a qualified resident physician; the third, the registration of births and deaths at the Union office instead of the police station. As headmen are aware, the existing statistics are far from reliable.

### Bombay Beggar Problem.

At a meeting of the Improvement Trust, the seriousness of the beggar problem in the city of Bombay was revealed. The Chairman stated that Mr. Manley, Deputy Commissioner of Police, wanted permission of the Board to use a part of the Dadar Military Camp for putting up temporary structures for housing homeless beggars. According to Mr. Manley, it was a perfect scandal that hundreds of people die in the streets, and a number of such deaths has been certified due to starvation. Influential members of the public had taken the matter up, and were discussing suitable means to tackle the problem. The Improvement Trust Board also agreed to giving the land asked for.

